# Development and validation of a discrimination model between primary PLA2R-negative membranous nephropathy and minimal change disease confirmed by renal biopsy

**DOI:** 10.1038/s41598-021-97517-8

**Published:** 2021-09-10

**Authors:** Feng Wu, Yiding Zhang, Wen Cui, Yijun Dong, Yingyang Geng, Changhao Liu, Zemeng Li, Yandong Xie, Xiaojing Cai, Jin Shang, Jing Xiao, Zhanzheng Zhao

**Affiliations:** Department of Nephrology, The First Affiliated Hospital, Zhengzhou University, No. 1, East Jianshe Road, Zhengzhou, 450052 Henan People’s Republic of China

**Keywords:** Diseases, Nephrology

## Abstract

Membranous nephropathy (MN) and minimal change disease (MCD) are two common causes leading to nephrotic syndrome (NS). They have similar clinical features but different treatment strategies and prognoses. M-type phospholipase A2 receptor (PLA2R) is considered as a specific marker of membranous nephropathy. However, its sensitivity is only about 70%. Therefore, there is a lack of effective and noninvasive tools to distinguish PLA2R-negative MN and MCD patients without renal biopsy. A total 949 patients who were pathologically diagnosed as idiopathic MN or MCD were enrolled in this study, including 805 idiopathic MN and 144 MCD. Based on the basic information and laboratory examination of 200 PLA2R-negative MN and 144 MCD, we used a univariate and multivariate logistic regression to select the relevant variables and develop a discrimination model. A novel model including age, albumin, urea, high density lipoprotein, C3 levels and red blood cell count was established for PLA2R-negative MN and MCD. The discrimination model has great differential capability (with an AUC of 0.904 in training group and an AUC of 0.886 in test group) and calibration capability. When testing in all 949 patients, our model also showed good discrimination ability for all idiopathic MN and MCD.

## Introduction

Membranous nephropathy (MN) is one of the most common glomerular diseases causing primary nephrotic syndrome (NS) in China. The prevalence of MN has increased greatly in Central China during the past two decades^1,2^. Minimal change disease (MCD) is another primary glomerular disease with high prevalence characterized by rapid onset and development. Current studies suggest these two diseases account for about 60% NS patients in China^3,4^.

Renal biopsy is still the gold standard for diagnosing and distinguishing these two diseases. However, considering the poor physical condition and contraindications, many patients are unsuitable to undergo renal biopsy in daily clinical practice^5^. In addition, it does take a period of time to wait for the result. The onset of MCD is usually utterly urgent which may cause irreversible damage to renal function if the treatment is not timely and correct.

M-type phospholipase A2 receptor (PLA2R) is considered as a specific marker of idiopathic membranous nephropathy. Present evidences reveal PLA2R test has strong specificity, while its sensitivity is insufficient, 0.7 approximately^6–9^. Hence, quite amount of PLA2R-negative MN patients cannot be screened out. More importantly, the accuracy of PLA2R detection may be influenced by other diseases such as tumor-related diseases^7,10^. Thrombospondin type-I domain-containing 7A (THSD7A) was identified as the target antigen in about 10% of patients with PLA2R-negative MN in European and North American populations, but THSD7A-associated membranous nephropathy has a low prevalence in Chinese patients^11^. Recently, multiple novel proteins/target antigens have been identified in MN, including exostosin 1/2, neural epidermal growth-like 1 protein, semaphorin 3B, protocadherin 7 and neural cell adhesion molecule 1^12,13^. Nevertheless, they are not still largely implemented in the routine clinical practice. Thus, it is urgent to develop a new convenient and noninvasive method to distinguish MN from MCD.

The aim of our study is to develop and validate a model to distinguish MCD and MN, especially PlA2R-negative MN. The model can be used to the patients who are unsuitable or unwilling to undergo renal biopsy. We believe our model will help clinicians treat these patients in a timely manner and improve their prognosis.

## Methods

### Study population and ethical approval

**Figure 1:**
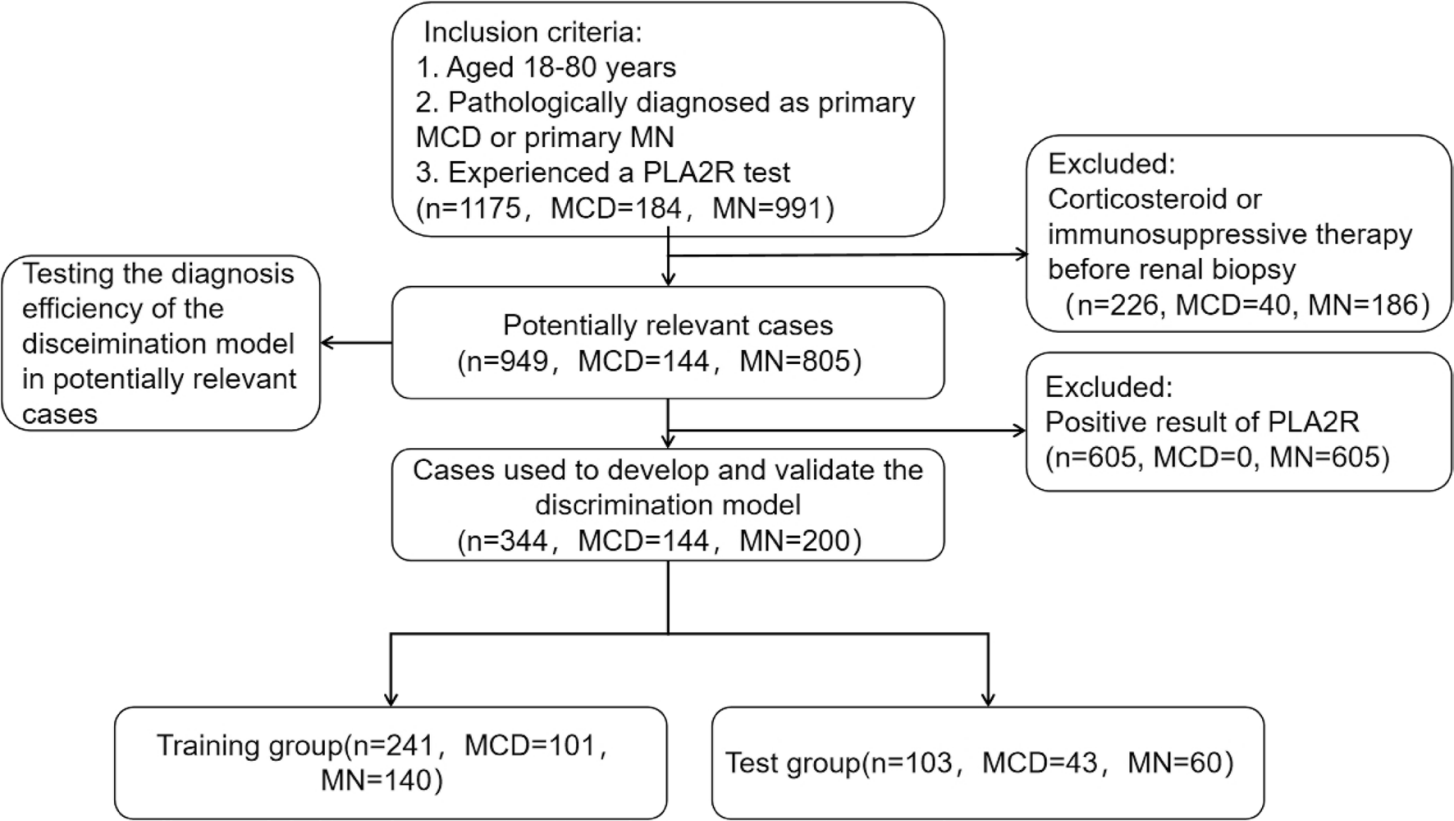
Enrollment flowchart of participants used for model development and validation. *MCD* minimal change disease, *MN* membranous nephropathy.

In this population-based retrospective analysis, we screened all the patients with NS who were hospitalized in the First Affiliated Hospital of Zhengzhou University from January 2017 to August 2019. The inclusion criteria were as follows: (1) aged 18–80 years, (2) diagnosed as idiopathic MN or primary MCD by renal biopsy, (3) experienced a PLA2R test. The exclusion criteria included application history of corticosteroid or immunosuppressant prior to renal biopsy. A total of 949 patients including 805 idiopathic MN (605 PLA2R-positive MN and 200 PLA2R-negative MN) and 144 primary MCD were enrolled in this study and defined as potentially relevant cases. Among them, patients with PLA2R-negative MN and MCD were used to develop and validate the discrimination model. In the end, we also tested the differential ability of the model in all idiopathic MN and MCD. Moreover, we also validated the model among 60 cases containing 42 PLA2R-negative MN and 18 primary NS caused by other etiology in real life (14 MCD, 3 focal segmental glomerulosclerosis, 1 mesangial capillary glomerulonephritis). Anti-PLA2R antibodies were determined by an ELISA assay. A negative serology was defined as an ELISA titer < 14 RU/mL. The enrollment flowchart of the participants in this study was shown in Fig. 1.

The First Affiliated Hospital of Zhengzhou University Ethics Review Committee granted ethical approval for the study and the ethics review approval ID was “ZY-2021-0008”, and the requirement for informed consent was waived by the ethics commission. All methods were performed in accordance with the relevant guidelines and regulations.

### Data collection

We collected the basic information and laboratory examination from all patients recruited at the time of renal biopsy, which might be involved in distinguishing the two diseases. The basic information included age, gender, onset time, systolic blood pressure (SBP) and diastolic blood pressure (DBP). The laboratory indices included red blood cell (RBC) count, white blood cell (WBC) count, platelet (PLT) count, eosinophil (EOS) count, percentage of eosinophils (EOS%), hemoglobin (Hb) levels, mean corpuscular hemoglobin (MCH), mean corpuscular hemoglobin concentration (MCHC), total protein (TP) levels, albumin (ALB) levels; total cholesterol (TC) levels, triglyceride (TC) levels, low density lipoprotein (LDL) levels, high density lipoprotein (HDL) levels, estimated glomerular filtration rate (eGFR); serum creatinine (SCr) levels; uric acid (UA) levels, C reactive protein (CRP) levels, erythrocyte sedimentation rate (ESR) levels, complement 3 (C3) levels, complement 4 (C4) levels, 24 h uric total protein (24 h TP) levels , 24 h urine volume and venous thrombosis of lower extremity.

The urine and venous blood samples of all participants were collected after 12 h of overnight fasting (except 24-h urine samples) and sent to laboratory for testing straight away.

### Statistical analysis

The PLA2R-negetive MN and all MCD patients (n = 344) were randomly divided into the training group (n = 241) and the test group (n = 103).

There were a few missing data of several variables. For example, the HLD and LDL levels had 3.5% missing values, Scr and Urea levels had 0.9% missing values. To deal with these missing data, multivariate multiple imputation with chained equations was used to impute missing values so that we could maximize the statistical power and diminish bias^14^. Descriptive statistics of all variables including means, medians, and proportions are used to describe the characteristics of two groups. The categorical data expressed as the percentages, and means ± standard deviation (SD) or medians (quartile 1, quartile 3) were described as continuous variables satisfying or not satisfying the normal distribution, respectively. We used the univariate logistic regression to calculate the OR values of the variables, selected potential variables to perform multiple logistic regression subsequently, and calculated the collinearity of the variables to remove the colinear factors. The candidate variables with a p value < 0.05 in the univariate analysis were enrolled to develop the multivariable model.

Based on the clinical features and variables with statistical sense, we attempted to develop the discrimination model for distinguishing PlA2R-negative MN from primary MCD patients.

Next, we drew a receiver operating characteristic curve (ROC) and used the area under the receiver operating characteristic curve (AUC) to evaluate the verification efficiency of the model. The calibration was assessed by constructing the calibration curve. The fitting degree of the model was assessed by the Akaike information criterion (AIC). After comprehensively evaluating the performance of each model, we obtained the best model and constructed a nomogram to make it convenient for the clinical application.

At last, we constructed the decision curve analysis to determine the clinical utility of the discrimination model by quantifying the net benefits at different threshold probabilities^15^.

All the statistical analysis processes involved were completed by R software, version 4.0.2. P value < 0.05 was considered statistically significant. Model validation and evaluation processes were independently performed on the training group and test group, respectively. In order to assess the diagnostic ability of the model distinguishing all idiopathic MN and MCD, we also performed validation processes on 949 potentially relevant cases.

### Ethics approval and consent to participate

The First Affiliated Hospital of Zhengzhou University Ethics Review Committee granted ethical approval for the study and the ethics review approval ID was “ZY-2021-0008”. Informed consent was waived because of the retrospective analysis.

## Results

### Baseline characteristics

The baseline characteristics of PLA2R-negative MN (n = 200) and MCD (n = 144) were shown in Table 1. The results suggested that PLA2R-negative MN patients tended to be older and have higher TP, ALB levels and greater urine volume. While, the RBC and PLT count, median Hb, TCHO, TG, LDL level, HLD, Scr, Urea, ESR, C3, C4 and 24hTP levels of PLA2R-negative MN were lower than those of MCD. The baseline characteristics of all idiopathic MN and MCD were shown in Table S1.

**Table 1:** Baseline characteristics of PLA2R-negative MN and MCD.

Variables	MN (n = 200)	MCD (n = 144)	p Value
Age (years)	50 (34, 58)	31 (24, 48)	< 0.001
Gender, n (male, %)	114 (57.0)	80 (55.6)	0.790
SBP (mmHg)	130 (120, 140)	127 (117, 135)	0.114
DBP (mmHg)	80 (76, 87)	83 (78, 90)	0.117
RBC (10^12^/L)	4.33 ± 0.58	4.64 ± 0.64	< 0.001
WBC (10^9^/L)	6.50 (4.50, 7.88)	6.2 (5.1, 7.6)	0.310
PLT (10^9^/L)	225 (192, 265)	252 (203, 313)	0.001
Eos (10^9^/L)	0.12 (0.08, 0.21)	0.13 (0.07, 0.23)	0.480
Eos%	2.00 (1.20, 3.00)	2.10 (1.37, 3.92)	0.230
Hb (g/L)	130.5 ± 18.1	138.9 ± 21.9	< 0.001
MCH (pg)	30.3 (29.3, 31.4)	30.4 (29.0, 31.4)	0.878
MCHC (g/L)	333 (327, 339)	333 (326, 338)	0.515
TP (g/L)	51.0 (44.7, 58.1)	41.2 (36.4, 46.8)	< 0.001
ALB (g/L)	28.05 (22.81, 34.38)	19.78 (16.83, 23.18)	< 0.001
TCHO (mmol/L)	6.05 (4.95, 7.83)	10.06 (7.86, 11.93)	< 0.001
TG (mmol/L)	1.85 (1.25, 2.67)	2.18 (1.51, 3.20)	0.018
LDL (mmol/L)	3.99 (3.11, 5.33)	7.82 (5.34, 9.66)	< 0.001
HDL (mmol/L)	1.25 (1.05, 1.57)	1.63 (1.29, 2.05)	< 0.001
eGFR (ml/min/1.73m^2^)	104.03 (90.24, 114.49)	100.16 (71.38, 118.85)	0.383
Scr (μmol/L)	68 (56, 80)	76 (61, 102)	< 0.001
Urea (mmol/L)	4.80 (3.73, 6.02)	5.50 (3.99, 8.38)	0.001
UA (μmol/L)	310.0 (259.3, 375.3)	317.5 (261.5, 385.3)	0.650
CRP (mg/L)	0.70 (0.05, 1.90)	0.83 (0.01, 2.02)	0.563
ESR (mm/h )	29.0 (16.8, 53.0)	55.0 (33.0, 77.2)	< 0.001
C3 (g/L)	1.29 (1.13, 1.47)	1.44 (1.26, 1.58)	< 0.001
C4 (g/L)	0.30 (0.25, 0.35)	0.33 (0.28, 0.38)	< 0.001
24hTP (g)	4.04 (1.92, 7.20)	6.43 (4.28, 9.04)	< 0.001
Urine volume (L)	1.6 (1.2, 2.2)	1.2 (0.7, 1.9)	< 0.001
Thrombosis, n (%)	1 (0.50)	5 (3.47)	0.086

*SBP* systolic blood pressure, *DBP* diastolic blood pressure, *RBC* red blood cell, *WBC* white blood cell, *PLT* platelet, *EOS* eosinophil, *Hb* hemoglobin, *MCH* mean corpuscular hemoglobin, *MCHC* mean corpuscular hemoglobin concentration, *TP* total protein, *ALB* albumin, *TC* total cholesterol, *TG* triglyceride, *LDL* low density lipoprotein, *HDL* high density lipoprotein, *eGFR* estimated glomerular filtration rate, *Scr* serum creatinine, *UA* uric acid, *CRP* C reactive protein, *ESR* erythrocyte sedimentation rate, *C3* complement 3, *C4* complement 4, *24hTP* 24 h uric total protein, *thrombosis* venous thrombosis of lower extremity.

All the participants were randomly divided into training group (n = 241) and test group (n = 103). The levels of these variables were similar and had no significant statistical difference, which represented similar clinical profiles between the two groups (Table 2).

**Table 2:** Baseline characteristics showed there was no statistical difference in the training group and test group.

Variables	Training group (n = 241)	Validation group (n = 103)	p Value
Age (years)	40 (29, 54)	48 (31, 55)	0.112
Gender, n (male, %)	131 (54.3)	63 (61.1)	0.244
SBP (mmHg)	130 (119, 139)	128 (120, 139)	0.745
DBP (mmHg)	81 (77, 90)	80 (76, 86)	0.278
RBC (10^12^/L)	4.47 ± 0.63	4.52 ± 0.61	0.706
WBC (10^9^/L)	6.50 (5.30, 7.90)	6.1 (5.2, 7.6)	0.268
PLT (10^9^/L)	234 (197, 279)	236 (186, 286)	0.694
Eos (10^9^/L)	0.14 (0.08, 0.24)	0.10 (0.06, 0.16)	0.002
Eos%	2.20 (1.40, 3.40)	1.90 (1.05, 2.70)	0.009
Hb (g/L)	134.4 ± 20.6	133.3 ± 19.3	0.648
MCH (pg)	30.4 (29.3, 31.5)	30.2 (29.0, 31.4)	0.863
MCHC (g/L)	333 (327, 340)	333 (327, 337)	0.459
TP (g/L)	46.6 (40.6, 54.4)	45.8 (39.7, 53.0)	0.648
ALB (g/L)	24.0 (19.5, 30.8)	22.9 (18.5, 31.0)	0.439
TCHO (mmol/L)	7.79 (5.70, 10.29)	6.90 (5.09, 9.64)	0.095
TG (mmol/L)	2.08 (1.36, 2.94)	1.85 (1.42, 2.50)	0.219
LDL (mmol/L)	5.14 (3.65, 7.88)	4.89 (3.31, 8.19)	0.472
HDL (mmol/L)	1.41 (1.12, 1.79)	1.32 (1.09, 1.78)	0.490
eGFR (ml/min/1.73 m^2^)	105.36 (88.83, 117.63)	98.96 (80.73, 110.67)	0.057
Scr (μmol/L)	69.0 (58.5, 84.5)	73.0 (60.0, 90.9)	0.141
Urea (mmol/L)	4.90 (3.80, 6.75)	5.00 (4.00, 6.60)	0.696
UA (μmol/L)	309 (254, 377)	324.0 (273, 395)	0.094
CRP (mg/L)	0.70 (0.00, 2.00)	1.00 (0.17, 2.04)	0.322
ESR (mm/h)	37.0 (20.0, 68.0)	44.0 (21.5, 68.0)	0.418
C3 (g/L)	1.37 (1.20, 1.56)	1.32 (1.13, 1.52)	0.159
C4 (g/L)	0.31 (0.25, 0.37)	0.32 (0.28, 0.37)	0.097
24hTP (g)	5.05 (2.38, 8.58)	5.16 (2.55, 7.81)	0.971
Urine volume (L)	1.5 (1.0, 2.1)	1.5 (1.0, 2.2)	0.787
Thrombosis, n (%)	4 (1.66)	2 (1.94)	1.000

### Six potential predictors were selected to develop the discrimination model

By means of univariate logistic regression, 17 potential predictors from 29 candidates were considered to have statistical significance (p < 0.05) in training group. After implementing the multivariable logistic regression analysis and removing the collinear candidates, six potential predictors including age, ALB levels, HDL levels, Urea levels, C3 levels and RBC counts were used to establish the discrimination model to distinguish PLA2R-negative MN from MCD (Table 3). We created a probability equation based on the above six predictors (Supplementary Equation). The results indicated that patients with older ages, higher ALB levels, lower HDL levels, lower serum Urea levels, lower C3 levels and lower RBC count were more likely suffering PLA2R-negative MN.

**Table 3:** Potential risk factors identified by univariate and multivariate logistic regression analysis.

Variable	Univariable		Multivariable	
	OR (95%CI)	P	OR (95%CI)	P
Age (years)	1.053 (1.033–1.073)	< 0.001	1.060 (1.026–1.095)	< 0.001
Gender, n (male, %)	0.927 (0.555–1.550)	0.773		
SBP (mmHg)	1.016 (1.000–1.032)	0.045		
DBP (mmHg)	0.987 (0.963–1.011)	0.288		
RBC (10^12^/L)	0.385 (0.243–0.611)	< 0.001	0.244 (0.071–0.846)	0.026
WBC (10^9^/L)	1.062 (0.932–1.210)	0.365		
PLT (10^9^/L)	0.993 (0.989–0.997)	0.001		
Eos (10^9^/L)	0.284 (0.073–1.104)	0.223		
Eos%	0.907 (0.824–0.999)	0.108		
Hb (g/L)	0.978 (0.965–0.991)	0.001		
MCH (pg)	1.036 (0.914–1.174)	0.582		
MCHC (g/L)	1.012 (0.988–1.038)	0.329		
TP (g/L)	1.116 (1.077–1.157)	< 0.001		
ALB (g/L)	1.174 (1.118–1.234)	< 0.001	1.183 (1.037–1.349)	0.012
TCHO (mmol/L)	0.656 (0.582–0.740)	< 0.001		
TG (mmol/L)	1.010 (0.893–1.142)	0.876		
LDL (mmol/L)	0.647 (0.570–0.734)	< 0.001		
HDL (mmol/L)	0.251 (0.142–0.445)	< 0.001	0.188 (0.083–0.426)	< 0.001
eGFR (ml/min/1.73m^2^)	1.007 (0.998–1.017)	0.122		
Scr (μmol/L)	0.992 (0.986–0.999)	0.016		
Urea (mmol/L)	0.851 (0.775–0.934)	0.001	0.777 (0.645–0.937)	0.008
UA (μmol/L)	0.999 (0.996–1.022)	0.501		
CRP (mg/L)	1.012 (0.983–1.041)	0.715		
ESR (mm/h)	0.982 (0.974–0.991)	< 0.001		
C3 (g/L)	0.201 (0.077–0.525)	0.001	0.088 (0.015–0.507)	0.007
C4 (g/L)	0.009 (0.001–0.140)	< 0.001		
24hTP (g)	0.865 (0.803–0.930)	< 0.001		
Urine volume (L)	1.945 (1.354–2.794)	< 0.001		
Thrombosis, n (%)	4.255 (0.436–41.513)	0.213		

### Great discrimination and calibration capability of the model

**Figure 2:**
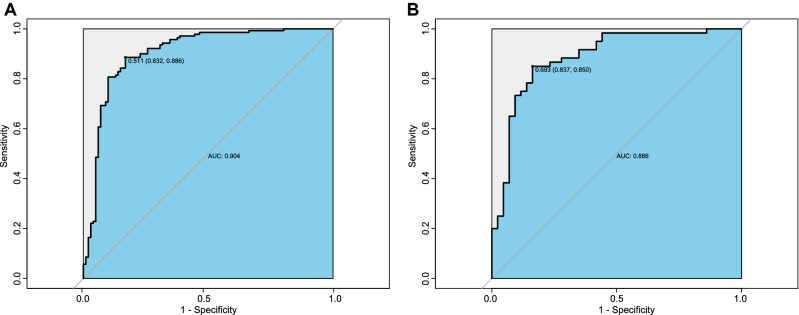
Differential capability of the nomogram. **(A)** ROC curve based on obtained potential risk factors identified by multivariate logistic regression analysis showing discrimination rate for PLA2R negative MN and MCD in the training group. **(B)** ROC curve based on obtained potential risk factors identified by multivariate logistic regression analysis showing discrimination rate for PLA2R negative MN and MCD in the test group.

We drew the ROC to evaluate the diagnostic effectiveness of the model (Fig. 2). The area under the ROC (AUC), which is referred to as the C-statistic, is considered to be an indicator for evaluating the effectiveness of the model. We found that the discrimination model had a high efficiency with an AUC of 0.904 (cut-off value: 0.511, sensitivity: 0.832, specificity: 0.886; Fig. 2A) in training group. We subsequently verified the effectiveness in the test group and the result showed a superior efficiency with an AUC of 0.886 (cut-off value: 0.693, sensitivity: 0.837, specificity: 0.850; Fig. 2B). The high value of AUC showed that the model had a great ability for discrimination the two diseases.

**Figure 3:**
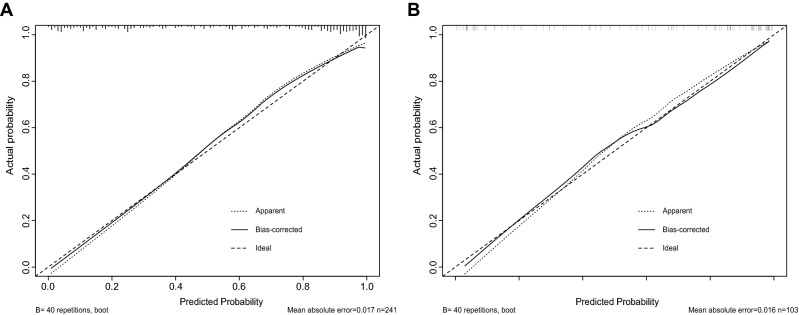
Calibration curve of the discrimination nomogram in the **(A)** training group or **(B)** test group. The x-axis represents the predicted probability of MN. The y-axis represents the actual pathologically diagnosed MN. The diagonal dotted line represents a perfect prediction by an ideal model. The solid line represents the performance of the nomogram, of which a closer fit to the diagonal dotted line represents a better prediction.

The calibration curve was plotted to evaluate the calibration of the model and it demonstrated a good agreement between prediction and observation both in training group (mean absolute error = 0.017) and test group (mean absolute error = 0.016, Fig. 3 A,B). The calibration curve indicated that the model had a great calibration capability.

### Construction and usage of the nomogram

**Figure 4:**
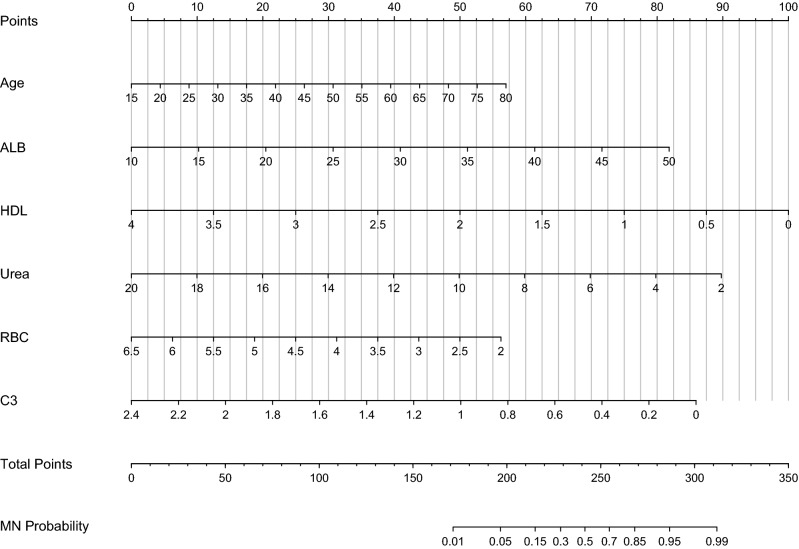
Nomogram based on the laboratory model; *ALB* albumin, *HDL* high density lipoprotein, *C3* complement 3, *RBC* red blood cell, *MN* membranous nephropathy.

In order to make the model convenient to use, we constructed the nomogram of our discrimination model based on six obtained predictive variables including age, HDL levels, ALB levels, Urea levels, C3 levels and RBC count (Fig. 4). The value of each variable represented as a score by drawing a straight line upward from the corresponding value to the “Points” line. Sum the total points and mark it at “Total points” line. Draw down a straight line to the corresponding “MN probability” axis and obtain the possibility of MN. In order to show the score intuitively, we have selected several representative values as examples (Table S2).

### Decision curve showed it would add more net benefits for clinical decision

**Figure 5:**
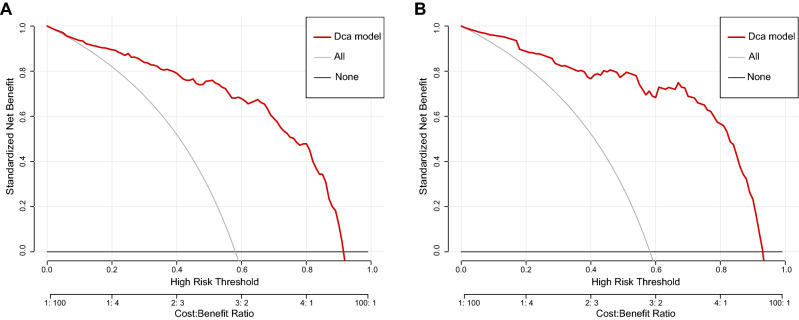
Decision curve for the nomogram predicting MN in **(A)** training group or **(B)** test group. Net benefit is shown on the y-axis. The thick red line represents the model; the thin gray line represents the assumption that all patients have MN; the thin black line represents the assumption that all patients have MCD. In training group, the decision curve showed that if the threshold probability of a patient is between 0.02 and 0.91, using the nomogram in the present study to predict MN adds more benefit than performing biopsy on all or no patients.

The result of decision curve analysis for the nomogram was shown in Fig. 5. In training group, the decision curve showed that if the threshold probability of a patient is between 0.02 and 0.91, using the nomogram in the present study to predict MN adds more benefits than performing renal biopsy on none or all patients.

### Diagnosis efficiency testing in potentially relevant cases

**Figure 6:**
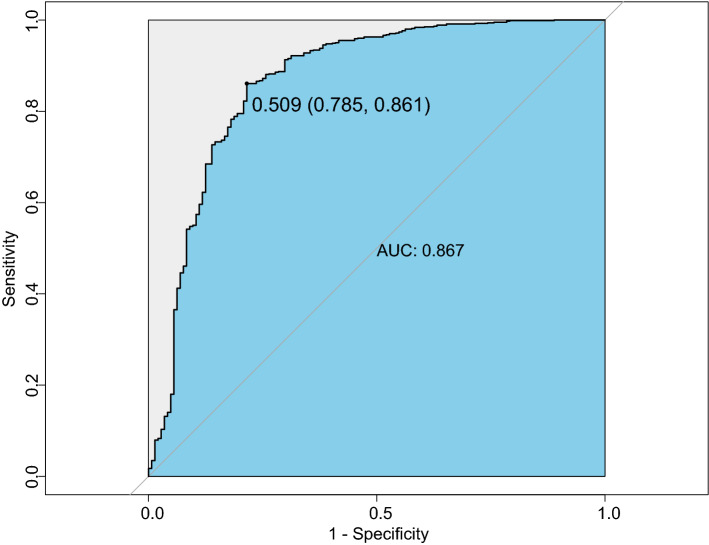
ROC curve showing discrimination effective of nomogram used in all MN and MCD.

**Figure 7:**
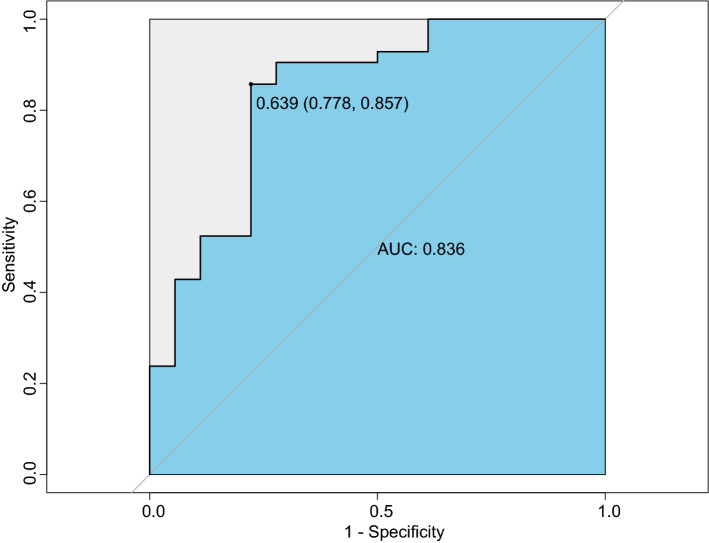
ROC curve showing discrimination effective of nomogram used in PLA2R-negative MN and primary NS caused by other etiology.

In order to determine whether we can expand the application scope of our model, we also performed diagnosis efficiency testing in potentially relevant cases (including 605 PLA2R-positive MN, 200 PLA2R-negative MN and 144 MCD, Fig. 6). The ROC showed the great diagnostic performance with an AUC of 0.867 (cut-off value: 0.509, sensitivity: 0.785, specificity: 0.861), suggesting great discrimination ability for all idiopathic MN and MCD patients. The performances of calibration curve and decision were also good (Figure S1, S2). In addition, we performed diagnosis efficiency test in PLA2R-negative MN and primary NS caused by other etiology (42 PLA2R-negative MN, 14 MCD, 3 focal segmental glomerulosclerosis, 1 mesangial capillary glomerulonephritis, Fig. 7). The ROC showed the diagnostic performance with an AUC of 0.836 (cut-off value: 0.639, sensitivity: 0.778, specificity: 0.857).

## Discussion

In this retrospective case–control study, we attempted to develop a discrimination model used to distinguish patients with idiopathic MN and MCD. We collected and analyzed the basic information and laboratory examination of 949 patients with MN or MCD. Based on 200 PLA2R negative patients and 144 MCD patients, we developed a discrimination model to differentiate the two diseases. The results showed great diagnostic effectiveness with an AUC of 0.904 in training group and an AUC of 0.886 in test group as well as high calibration capability. To the best of our knowledge, it is the first study aiming to develop a discrimination model based on the basic information and the laboratory examination of participants to distinguish primary PLA2R-negative MN and MCD. In addition, our model showed great diagnostic effectiveness with an AUC of 0.867 in all idiopathic MN patients (either PLA2R-negative or PLA2R-positive MN) and MCD patients. It is an attempt at translational medicine of our study, which can aid clinicians to treat patients with different methods in a timely manner and thus improve their prognosis.

Currently, it is difficult to distinguish MN and MCD patients by a noninvasive tool in clinical practice. A study tried to use soluble urokinase-type plasminogen activator receptor (suPAR) level to distinguish idiopathic focal segmental glomerulosclerosis (FSGS), MN and MCD. However, the study revealed that the three types of glomerulopathy cannot be distinguished using suPAR solely ^16^. Therefore, there is no miraculous indicator or model to identify these two primary glomerular diseases currently.

Prediction and discrimination models based on clinical data have been developed increasingly in a wide variety of diseases recent years^17,18^. In terms of kidney diseases, prediction and discrimination models are also rapidly growing owing to its scientific nature and accuracy^19–21^. The appearance of clinical models gave us great inspiration.

Present evidences suggested that MN had the largest proportion of morbidity in elderly patients, while MCD accounts for the highest proportion of primary nephrotic syndrome in young patients^3,22,23^. Our experiments reached similar results that the age at biopsy had a certain influence on the nomogram. In our study, patients with older age were more likely to be considered as PLA2R-negative MN, while younger age at onset was considered to be a higher risk of MCD.

ALB level was one of the predictive factors in this model. Some studies showed MN patients always had higher ALB levels, which was consistent with our results^24,25^. One of the most important clinical manifestations of nephrotic syndrome is increased urinary protein and decreased albumin level. Larger amounts of glomerular albumin filtration will also make serum albumin and serum total protein at a low level. MCD patients always presents as an acute onset, severe illness, and a greater amount of urinary protein, and rapid decline in renal function may result in increased Scr levels and decreased eGFR and 24 h urine volume. Some patients will even progresses to AKI, which is relatively common actually, due to high-grade proteinuria^26^. The slit diaphragm between foot processes is regarded as a fine filter^27^. There is a common assumption that proteins leak from the slit pores due to reduced nephrin expression, leading to larger amounts of glomerular albumin filtration in MCD patients^28^. In our study, we chose ALB levels as one of the variables of the model by multivariable regression analysis to exclude the effect of collinearity.

HDL level is another variable that can be used to distinguish between two diseases according to our results. Nephrotic syndrome could cause upregulation of HDL endocytic receptor and downregulation of HDL docking receptor, causing dysregulation of lipid/lipoprotein metabolism^29^. MCD is also known as lipoid nephropathy because steatosis can be observed in epithelial cells of proximal convoluted tubules under light microscopy. In addition, increased hepatic lipoprotein synthesis and reduced lipoprotein degradation are also thought to be responsible for elevated blood lipid profiles. Takeshi Fujita compared lipid and fatty acid metabolism between 7 MCD and 11 MN patients. The results showed that the patients with MCD had higher level of blood lipids than MN^30^. Although the mean HDL level was much higher in patients with MCD, there was no statistical significance between the two groups, which was not exactly the same as our results. The reason might be that their sample size was not large enough, leading to the unobvious statistical significance.

Increased urea level was usually observed in a high protein decomposition status. After the renal filtration barrier disrupted, large amount of protein will leak into Bowman’s space and renal tubules through glomerular barrier to form crude urine. When proximal tubules enhance the reabsorption of filtered proteins, the protein decomposition is also increased at the same time, resulting in elevated serum Urea level. Serum Urea level were also found different in MN patients and MCD patients in Jin Dong’s research^31^, which was consistent with our results. The Urea level plays an important role in our discrimination model.

C3 level is also a biomarker for distinguishing PLA2R-negative MN and MCD. Complement is a group of glycoproteins with enzyme-like activity that exists in human serum and tissue fluid, together with its regulatory factors and related membrane proteins to form the complement system^32^. C3 is the largest content in each component of the complement system and the key substance of the classical pathway and the alternative pathway. A variety of glomerulonephritis showed evidence of complement activation. However, the role of complement in the pathogenesis of these kidney diseases remains not fully understood^33^. From the study of combining histopathological examination and blood test to identify different types of glomerulonephritis, the C3 level of MCD is higher than that of MN^31^, although there is no statistical significance which may be due to insufficient cases.

RBC count and Hb levels had statistical significance by means of univariate regression in our study. They are usually recognized as the indices to evaluate anemia. Compared with younger patients, idiopathic membranous nephropathy patients over 65 years old were found to have lower Hb level than patients less than 65 years old in Choi JY’s study^34^. However, the results in Yaeni Kim’s study showed there was no difference of Hb levels between elderly patients and young patients^35^. The reason for this ambiguity might be different gender and illness state of included patients. In our study, univariate regression showed there was no statistical difference in gender. And the data we collected was from the time of renal biopsy, reducing influence of the illness state.

The decision curve showed the clinical utility of our model, indicating it may be beneficial for clinicians to distinguish the two diseases by using our model. And using the nomogram to distinguish the two diseases added more benefits than either all or no patients who underwent a renal biopsy if the threshold probability of a patient was between 0.02 and 0.91. The results of decision curves suggest the good clinical application value of our model, reflecting the thinking mode of translational medicine.

The results of diagnosis efficiency test in potentially relevant cases suggested that our model is applicable to all idiopathic MN and MCD patients. Some hospitals are unable to perform PLA2R test, and our model might provide an alternative tool for these hospitals to distinguish MN and MCD.

Our study is an attempt in translational medicine and has a number of strengths. It had a large sample size with 949 idiopathic MN and MCD patients confirmed by renal biopsy. And the 6 items in the nomogram are routine clinical variables that can easily obtained by clinicians. We chose to collect the information and examination results at the time of renal biopsy, and excluded the influence of corticosteroid or immunosuppressive agents. What is more, our discrimination model has excellent diagnostic effectiveness with an AUC of 0.904 in training group and an AUC of 0.886 in test group. The outstanding discrimination ability for all idiopathic MN and MCD patients even showed wider application prospects of our model. The operation of the model is simple and fast, which can help doctors diagnose patients timely. Unlike renal biopsy, our model does not have any contraindications so that it can be used more widely.

However, there are also several limitations in our study. First, we still need to expand the sample size for further reducing the heterogeneity. In addition, all the patients came from the First Affiliated Hospital of Zhengzhou University and we did not conduct multicenter external validation. Besides, the parameters of our model mainly came from laboratory test (e.g. red blood cell count and albumin). These parameters are non-specific and may be affected by many factors, which is one of our limitations. Last, our model is only suitable for the identification of MN and MCD. The incidence of MN and MCD is high, while IgA nephropathy is the most common pattern of primary glomerular disease worldwide. Even through IgA nephropathy always presents the clinical features as nephritis with minor proteinuria rather than nephrotic syndrome, unlike MN and MCD^3,36^, there is still a lack of differential ability of our model for other types of nephrotic syndrome. Corrections to these shortcomings will be made in our subsequent research.

## Conclusion

In this study, we developed and validated a discrimination model used for distinguishing PLA2R-negative MN and MCD patients. We further presented a nomogram including age, ALB levels, HDL levels, urea levels, C3 levels and RBC counts. The model showed good discrimination and calibration ability both in training group and test group. It also had a great diagnostic performance in all MN patients and MCD patients. Hopefully, it could provide a practical and convenient tool for clinicians to distinguish these two diseases.

## Supplementary Information

Supplementary Information.
