# Supplementary material for: Development and validation of a discrimination model between primary PLA2R-negative membranous nephropathy and minimal change disease confirmed by renal biopsy
Source: Sci Rep. 2021 Sep 10;11:18064. doi: 10.1038/s41598-021-97517-8 (PMC8433159; doi:10.1038/s41598-021-97517-8)
Supplement: Supplementary file 1 — Supplementary Information. [file 41598_2021_97517_MOESM1_ESM.docx]

**Supplementary materials**

**Supplementary Equation**：

$$P=\frac{\exp(A)}{1+exp(A)}$$

A=5.99606+0.05734×Age+0.13378×ALB-1.63475×HDL-0.32614×Urea-0.81712×RBC-2.34113×C3；

The best cut-off value of the probability score P is 0.511. When the probability score of the patient is lower than 0.511, the probability of being MN is low, and the result of renal biopsy of the patient is more likely to be MCD. When the patient's probability score is higher than or equal to 0.511, the patient's renal biopsy result is highly likely to be MN.

**Figure S1**

**
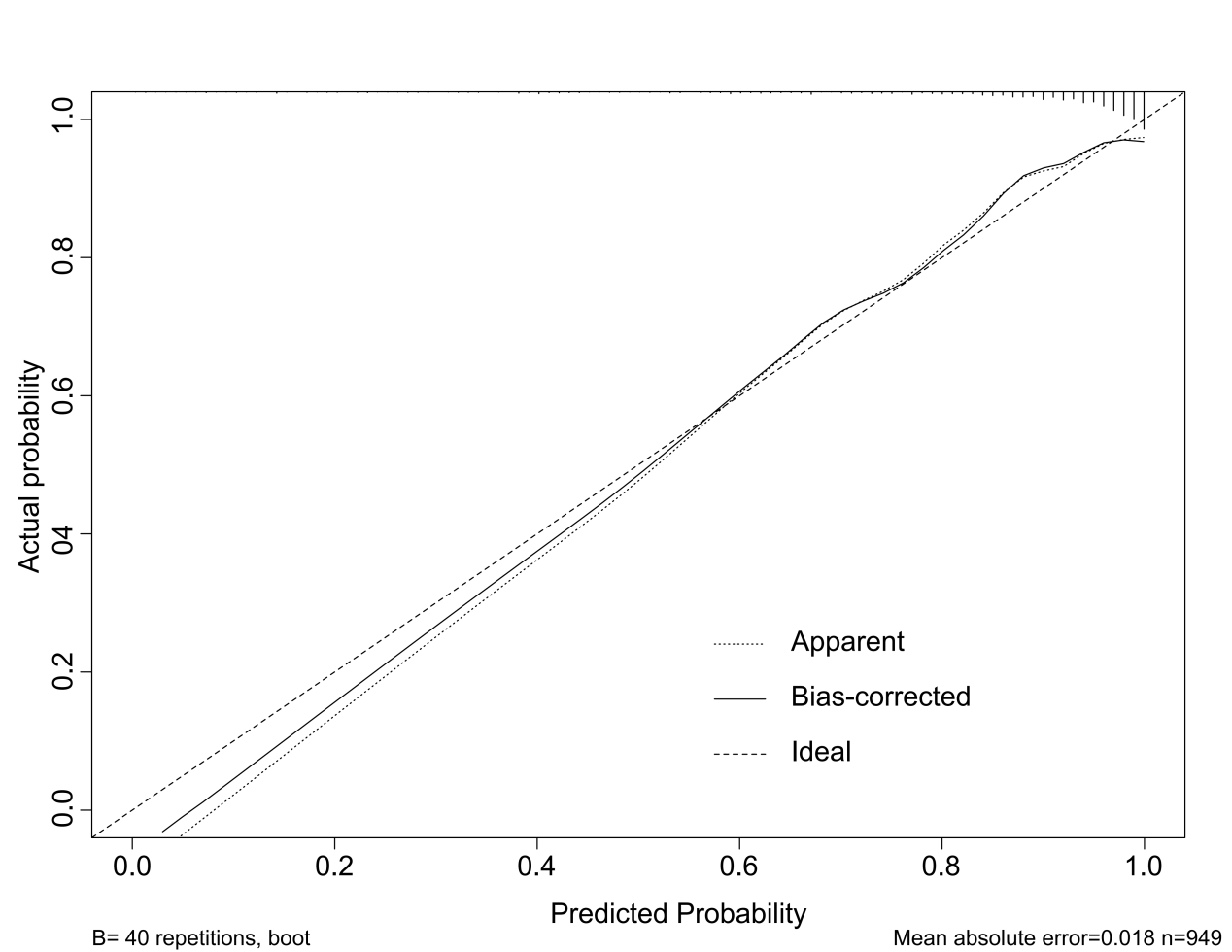
**Figure S1. Calibration curve of the discrimination nomogram used in potentially relevant MN and MCD.

**Figure S2**


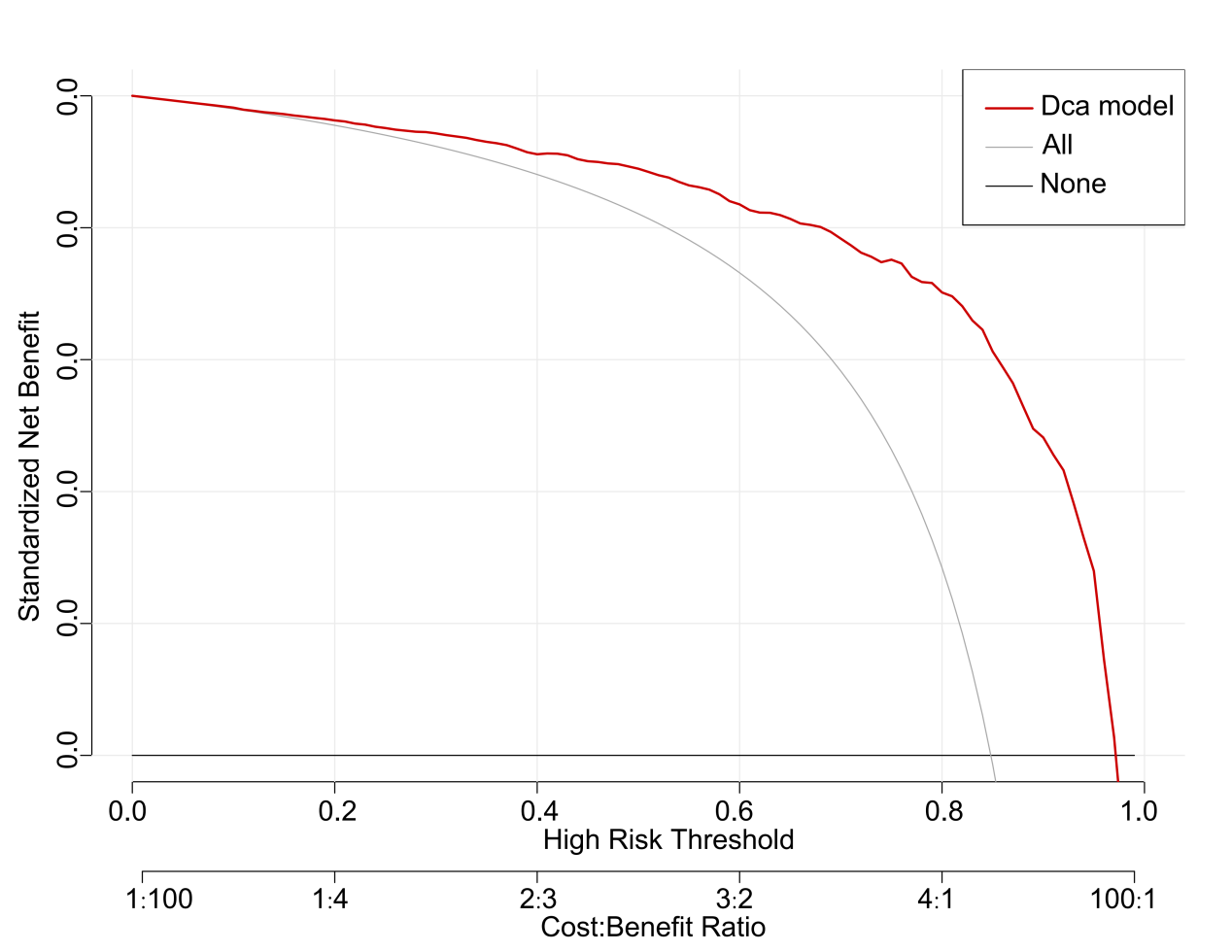
Figure S2. Decision curve for the nomogram predicting MN in potentially relevant cases.

| **Table S1** |  |  |  |
| --- | --- | --- | --- |
| **Variables** | **MN (n=805)** | **MCD (n=144)** | **p Value** |
| Age (years) | 49(38,58) | 31(24,48) | <0.001 |
| Gender, n (male, %) | 503(62.5) | 80(55.6) | 0.116 |
| SBP (mmHg) | 131(122,142) | 127(117,135) | <0.000 |
| DBP (mmHg) | 83(78,90) | 83(78,90) | 0.899 |
| RBC (10^12^/L) | 4.35±0.59 | 4.63±0.64 | <0.001 |
| WBC (10^9^/L) | 6.6(5.4,7.9) | 6.2(5.1,7.6) | 0.089 |
| PLT (10^9^/L) | 234(196,278) | 252(203,313) | 0.008 |
| Eos(10^9^/L) | 0.12 (0.06，0.21) | 0.12 (0.06，0.20) | 0.841 |
| Eos% | 1.90 (1.00，3.20) | 1.90 (1.08，3.62) | 0.545 |
| Hb (g/L) | 131.5±18.7 | 138.9±21.9 | <0.001 |
| MCH (pg) | 30.3(29.3,31.3) | 30.4(29.0,31.4) | 0.851 |
| MCHC (g/L) | 333(328,339) | 333(326,338) | 0.375 |
| TP (g/L) | 46.5(40.9,52.6) | 41.2(36.4,46.8) | <0.001 |
| ALB (g/L) | 24.6(20.7,29.3) | 19.8(16.8,23.2) | <0.001 |
| TCHO (mmol/L) | 7.09(5.64,8.79) | 10.06(7.86,11.93) | <0.001 |
| TG (mmol/L) | 2.00(1.36,2.93) | 2.18(1.51,3.20) | 0.129 |
| LDL (mmol/L) | 4.92(3.63,6.60) | 7.82(5.34,9.66) | <0.001 |
| HDL (mmol/L) | 1.29(1.07,1.65) | 1.63(1.29,2.05) | <0.001 |
| eGFR (ml/min/1.73m^2^) | 101.70(86.95,112.33) | 100.16(71.38,118.85) | 0.789 |
| Scr (μmol/L) | 70(59,85) | 76(61,102) | <0.001 |
| Urea (mmol/L) | 4.9(4.0,6.2) | 5.5(4.0,8.4) | 0.001 |
| UA (μmol/L) | 312.0(263.5,373.5) | 317.5(261.5,385.3) | 0.750 |
| CRP(mg/L) | 0.80 (0.13，2.10) | 0.83 (0.00，2.15) | 0.926 |
| ESR(mm/h ) | 30.0 (16.0，50.0) | 55.0 (30.0，80.0) | <0.001 |
| C3(g/L) | 1.29 (1.12，1.47) | 1.44 (1.26，1.58) | <0.001 |
| C4(g/L) | 0.31 (0.26，0.36) | 0.33 (0.28，0.38) | 0.001 |
| 24hTP (g) | 5.69(3.10,8.50) | 6.43(4.28,9.04) | 0.010 |
| Urine volume (L) | 1.5(1.1,2.0) | 1.2(0.7,1.9) | <0.000 |
| Thrombosis, n (%) | 19 (2.36%) | 5 (3.47%) | 0.393 |

Table S1. Baseline characteristics of all MN and MCD.

**Table S2**

|  | 1 | 2 | 3 | 4 | 5 | 6 |
| --- | --- | --- | --- | --- | --- | --- |
| Age | 20 | 30 | 40 | 50 | 60 | 70 |
| point | 4 | 13 | 22 | 31 | 49 | 48 |
| ALB/ g/l | 15 | 20 | 25 | 30 | 40 | 50 |
| point | 10 | 20 | 31 | 41 | 61 | 82 |
| HDL/ mmol/l | 0.5 | 1.0 | 1.5 | 2.0 | 2.5 | 3.0 |
| point | 88 | 75 | 63 | 50 | 38 | 25 |
| Urea/mmol/l | 4 | 6 | 8 | 10 | 12 | 14 |
| point | 80 | 70 | 60 | 50 | 40 | 30 |
| RBC/10^12/L | 2.5 | 3.0 | 3.5 | 4.0 | 4.5 | 5.0 |
| point | 50 | 44 | 37 | 31 | 25 | 19 |
| C3/g/L | 0.2 | 0.6 | 1.0 | 1.6 | 2.0 | 2.2 |
| point | 79 | 64 | 50 | 29 | 14 | 7 |
| Total point | 171 | 215 | 229 | 242 | 268 | 312 |
| MN Probability | 0.01 | 0.15 | 0.30 | 0.50 | 0.85 | 0.99 |

Table S2. Examples of specific scores. ALB, Albumin; HDL, High density lipoprotein; RBC, Red blood cell. MN, Membranous nephropathy.
